# Effects of proteome rebalancing and sulfur nutrition on the accumulation of methionine rich δ-zein in transgenic soybeans

**DOI:** 10.3389/fpls.2014.00633

**Published:** 2014-11-11

**Authors:** Won-Seok Kim, Joseph M. Jez, Hari B. Krishnan

**Affiliations:** ^1^Plant Genetics Research Unit, Agricultural Research Service, U.S. Department of Agriculture, University of MissouriColumbia, MO, USA; ^2^Department of Biology, Washington UniversitySt. Louis, MO, USA

**Keywords:** *Glycine max* (L.) Merr., 7S globulin, RNA interference, proteome rebalancing, sulfur assimilation, δ-zein

## Abstract

Expression of heterologous methionine-rich proteins to increase the overall sulfur amino acid content of soybean seeds has been only marginally successful, presumably due to low accumulation of transgenes in soybeans or due to gene silencing. Proteome rebalancing of seed proteins has been shown to promote the accumulation of foreign proteins. In this study, we have utilized RNAi technology to suppress the expression of the β-conglycinin, the abundant 7S seed storage proteins of soybean. Western blot and 2D-gel analysis revealed that β-conglycinin knockdown line (SAM) failed to accumulate the α′, α, and β-subunits of β-conglycinin. The proteome rebalanced SAM retained the overall protein and oil content similar to that of wild-type soybean. We also generated transgenic soybean lines expressing methionine-rich 11 kDa δ-zein under the control of either the glycinin or β-conglycinin promoter. The introgression of the 11 kDa δ-zein into β-conglycinin knockdown line did not enhance the accumulation of the 11 kDa δ-zein. However, when the same plants were grown in sulfur-rich medium, we observed 3- to 16-fold increased accumulation of the 11 kDa δ-zein. Transmission electron microscopy observation revealed that seeds grown in sulfur-rich medium contained numerous endoplasmic reticulum derived protein bodies. Our findings suggest that sulfur availability, not proteome rebalancing, is needed for high-level accumulation of heterologous methionine-rich proteins in soybean seeds.

## Introduction

Due to their high protein content and relatively low cost, soybeans (*Glycine max* (L.) Merr.) are used as an animal feed throughout the world. Protein accounts for about 40% of the dry weight of soybean seeds. The abundant storage proteins of soybean are the salt-soluble globulins, the 7S β-conglycinin and the 11S glycinin, which together could account for about 70% of the total seed proteins (Nielsen, 1996; Nielsen and Nam, 1999; Krishnan, 2000). Both these classes of proteins are encoded by multiple gene families (Harada et al., 1989; Nielsen et al., 1989). Glycinins are hexameric proteins that are classified into two groups based on DNA sequence similarities (Nielsen et al., 1989). Group-1 glycinins contain three genes, *Gy1*, *Gy2*, and *Gy3*, while group-2 glycinins include two genes, *Gy4* and *Gy5*. In addition, two genes, *Gy7* and *Gy6*, resembling group-1 glycinins are also present (Beilinson et al., 2002). The expression of *Gy7* gene is significantly lower than other *Gy* genes while the Gy6 has been identified as a pseudogene (Beilinson et al., 2002). Each of the glycinins is synthesized as a precursor protein that is subsequently cleaved into acidic and basic subunits (Staswick et al., 1984). β-conglycinin consists α′, α, and β subunits (Coates et al., 1985). Unlike the glycinins, the β-conglycinins are glycoproteins (Thanh and Shibasaki, 1978). Because of their abundance, the 7S and 11S globulins are mainly responsible for the nutritional quality of soybeans.

Monogastric animals including humans are unable to synthesize essential amino acids and are dependent on their feed/food to meet the essential amino acid requirements. Even though soybeans are an excellent source of high quality protein, the sulfur amino acid content of soybean seed proteins is not optimal for the formulation of animal feed. This deficiency necessitates supplementation of animal feeds with synthetic methionine that adds additional cost to the livestock producers. Consequently, there have been numerous attempts to improve the sulfur amino acid content of legume seed proteins utilizing different approaches including genetic engineering (Tabe and Higgins, 1998; Krishnan, 2005; Amir et al., 2012; Galili and Amir, 2013). One common approach involves the expression of heterologous seed proteins in transgenic soybeans (Townsend and Thomas, 1994; Dinkins et al., 2001; Kim and Krishnan, 2004; Krishnan, 2005). We have previously reported the expression a methionine-rich δ-zein in soybean (Kim and Krishnan, 2004). The δ-zein stably accumulated with endoplasmic derived protein bodies in transgenic soybean seeds. However, the accumulation of the δ-zein was less than 0.5% of the total seed protein and did not increase the overall methionine content of soybean seed (Kim and Krishnan, 2004). Similarly, attempts by others to substantially increase the methionine content of soybeans by expressing heterologous proteins rich in methionine have not been successful (reviewed in Krishnan, 2005). Low gene expression of methionine-rich protein in soybeans can be a major contributing factor for the marginal increase in the methionine content in transgenic soybeans.

Several approaches to increase the amount of heterologous protein production in plants have been proposed (Streatfield, 2007). These approaches focus on boosting the heterologous gene replication, transcription, and translation and message stabilization (Streatfield, 2007). Another approach to enhance foreign protein production exploits the high protein synthesis capacity of legumes such as soybean (Schmidt and Herman, 2008; Herman, 2014). By suppressing the production of endogenous seed storage proteins, one could redirect the available protein synthesis capacity to the synthesis of introduced foreign proteins. To test this hypothesis, a green fluorescent protein (GFP)-kdel reporter was introgressed in β-conglycinin suppressed transgenic soybeans. This approach resulted in four-fold increase in GFP-kdel accumulation in transgenic soybean suggesting that proteome rebalancing can enhance foreign protein accumulation (Schmidt and Herman, 2008). Since our previous attempt to increase the sulfur amino acid content of soybean seed by expressing methionine-rich δ-zein was marginally successful due to low accumulation of the δ-zein, we wanted to explore if proteome rebalancing could be exploited to elevate the δ-zein accumulation in soybean.

In this study, we have created β-conglycinin knockdown transgenic soybean lines expressing methionine-rich 11 kDa δ-zein. Interestingly, the expression the 11 kDa δ-zein in β-conglycinin knockdown lines did not elevate the accumulation of the methionine-rich protein. However, when the same plants were grown in sulfur-rich medium, a drastic increase in the 11 kDa δ-zein accumulation was observed. Our results indicate that the availability of sulfur, not proteome rebalancing, is more critical for high-level accumulation of methionine-rich proteins in soybean seeds.

## Materials and methods

### Generation of B-conglycinin suppressed transgenic soybeans

**Figure 1:**
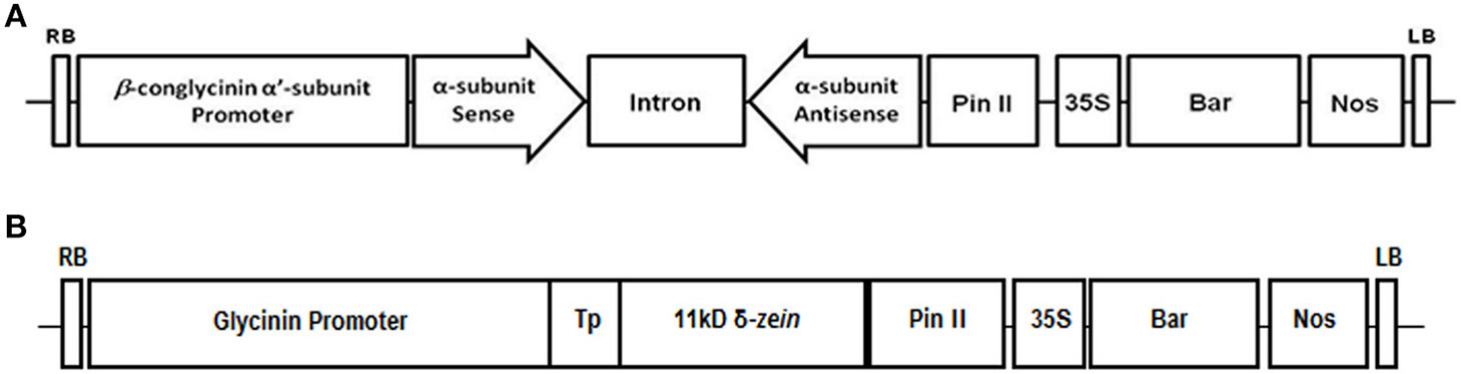
**Schematic diagram of the constructs used for the suppression of β-conglycinin soybean seeds (A) and expression of 11 kDa δ-zein (B)**. The RNAi cassette contains a 314 bp region of the α′ subunit of the β-conglycinin cloned in an inverted repeat orientation and separated by the intron from pKannibal. The RNAi construct is under the control of the soybean β-conglycinin α′-promoter while the expression of 11 kDa δ-zein is under the control of soybean glycinin promoter (Kim and Krishnan, 2004). The constructs also contain a gene expression cassette that includes the cauliflower mosaic virus 35S promoter, the bar-coding region and the 3′ region of the nopaline synthase gene (nos).

To simultaneously suppress the expression of all three subunits of β-conglycinin we constructed an RNAi cassette by the following procedure. First, the intron from pKannibal (Wesley et al., 2001) was amplified employing the primer pair: 5′ primer *XbNdXh*INTRi (5′-**TCTAGA** A**CATATG**GTC**CTCGAG**AGTTACTAGTACCCCA-3′) containing *Xba*I, *Nde*I and *Xho*I sites (bold) and 3′ primer INTRi*RVSXb* (5-**TCTAGA**G**GTCGAC**CG**GATATC**CGCTTTGTTATATTAGC-3) containing XbaI, *Sal*I, and *Eco*RV sites (bold). The amplified intron product was cloned into pGEM-T easy to create pKINTRi. The intron was excised by digestion with *Xba*I and cloned into pHK vector. Primer pair SNd2AplhaRi (5′-GTCGAC**CATATG**TACAGGAACCAAGCATGCCAC-3′; introduced restriction sites *Sal*I and *Nde*I are underlined and bold, respectively) and Alpha′314XhRV (5′-GATATC**CTCGAG**TGAGGTTGGTGTGGGCGTGGG-3′; introduced restriction sites *EcoRV* and *Xho*I are underlined and bold, respectively) was used to amplify a 314 bp coding region of α′-subunit of the β-conglycinin. This 314 bp product was cloned into pGEM-Teasy vector (Promega, WI) and named as pBCON and sequenced at the University of Missouri DNA Core Facility. DNA prepared from pBCON was subjected to restriction enzyme digests with *Nde*I and *Xho*I or *Eco*RV and *Sal*I to facilitate cloning the 314 bp fragments in the opposite orientation. The *Nde*I and *Xho*I excised fragment was ligated into the 5′ end of the pHK-intron while the *Eco*RV and *Sal*I fragement was ligated into the 3′ end pHK-intron. This procedure resulted in a cassette containing a 314 bp α′-subunit of the β-conglycinin hairpin flanking the pHK-intron. Following digestion with *Xba*I the entire β-conglycinin hairpin was cloned into the corresponding restriction site of pZPlapha′P binary vector (Kim and Krishnan, 2004). This final vector places the β-conglycinin hairpin under the regulatory control of the α′-subunit of β-conglycinin promoter and the terminator of the potato proteinase inhibitor gene (*Pin*II). This vector also contains the *bar*-coding region under the regulatory control of cauliflower mosaic virus 35S promoter and 3′-region of the nopaline synthase gene (Figure 1). The final RNAi construct was mobilized into *Agrobacterium tumefaciens* (strain EHA105) by triparental mating (Friedman et al., 1982). Soybean cultivar Maverick was transformed by *Agrobacterium*-cotyledonary node method (Hinchee et al., 1988). Regenerated transgenic soybean plants were screened for tolerance to herbicide Liberty by a leaf-painting assay as described earlier (Zhang et al., 1999). The knockout of the target proteins in glufosinate-resistance plants was confirmed by PCR and SDS-PAGE. For all comparisons soybean cultivar Maverick was used and referred as wild-type.

### RT-PCR analysis

Total RNA was extracted from transgenic soybean seed at the mid-maturation stage (seed size 8–10 mm) using a Trizol reagent (Invitrogen, Grand Island, NY, USA) following the manufacture's protocol. RNA was quantified by measuring the A260/A280 ratio using a spectrophotometer. One μg of DNase I treated RNA was used as template for RT-PCR. Primers used for alpha′ subunit were Alpha′F, 5-ATGATGAGAGCGCGGTTCCCATTACTG-3 and Alpha′R, 5-TCAGTAAAAAGCCCTCAAAATTGAAGAC-3. Primers used for alpha subunit were Alpha F, 5-ATGATGAGAGCACGGTTCCCATTACTG-3 and Alpha′R. Primers used for beta subunit were BetaF, 5-ATGATGAGAGTGCGGTTTCCTTTGTTGG-3 and BetaR, 5- TCAGTAGAGAGCACCTAAGATTGAAG-3. Primers used for glycinin 4 were SoyGy4F, 5- ATGGGGAAGCCCTTCACTCTCTCTCTTTC-3 and SoyGy4R, 5- TTATGCGACTTTAACACGGGGTGAGC-3. The cycling condition of RT-PCR were 50°C for 30 min for cDNA production, 95°C for 15 min for inactivation of cDNA production, then 35 cycles of 94°C for 60 s, 60°C for 60 s, 72°C, for 90 s with a final 72°C for 240 s extension steps.

### Determination of seed protein and oil content

Soybean protein content was measured using the Leco model FP-428 nitrogen analyzer (LECO Corporation, Michigan, USA). The oil content was quantified by near-infrared reflectance (NIR) spectroscopy (Tecator AB, Hoganas, Sweden). The fatty acid profiles of soybean were determined by gas chromatograph as described previously (Lee et al., 2009). Briefly, crushed seeds were extracted overnight with 5 mL of chloroform: hexane: methanol (8:5:2, v/v/v). Fatty acids from 100 μL aliquots of the extract were methylated with 75 μL of methanolic sodium methoxide:petroleum ether:ethyl ether (1:5:2, v/v/v). Fatty acids were separated utilizing Agilent Series 6890 capillary gas chromatograph (Palo Alto, CA, USA) that was fitted with an AT-Silar capillary column (Alltech Associates, Deerfield, IL, USA). Standard fatty acid mixtures were used for determining relative amounts of each fatty acid. Four replicates of “Maverick” (MAV) and β-conglycinin knockdown line (SAM) were compared using the t test function within JMP® software Version 9 (SAS Institute Inc., Cary, NC). Significantly different means are indicated by ^*^, ^**^, or ^***^ (*p* ≤ 0.05, *p* ≤ 0.01, *p* ≤ 0.001, respectively) and insignificant differences are indicated by “NS.”

### Protein isolation and immunoblot analysis

Total soybean seed proteins were extracted from 10 mg of dry seed powder with 1 ml of SDS sample extract buffer (2% SDS, 60 mM Tris-HCl, pH 6.8, 5% β-mecaptoethanol), followed by boiling at 100°C for 5 min. After maximum speed centrifuge the supernatant was used for total seed protein fraction. The total seed protein fraction was electrophoresed with 8 or 15% SDS-PAGE and visualized by staining with Coomassie Brilliant Blue. For western blot analysis, the total seed proteins were transferred to a nitrocellulose membrane after resolved by SDS-PAGE. After blocking with TBS (10 mM Tris-HCl, pH 7.5, 500 mM NaCl) containing 5% non-fat dry milk, the membrane was incubated over-night with 7S globulin storage protein or the 11 kDa δ-zein antibodies (Kim and Krishnan, 2003) that had been diluted 1:5000 in TBST (TBS with 3% non-fat dry milk containing 0.2% Tween 20). After several washes with TBST, the membrane was incubated with goat anti-rabbit IgG-horseradish peroxidase conjugate. Immunoreactive polypeptide signals were detected by according to the SuperSignal West Pico kit's instruction (Pierce, Rockford, Il, USA).

### Two-dimensional gel electrophoresis, image analysis and quantification of spot volume

Two-dimensional gel electrophoresis of soybean seed proteins were performed as described earlier (Krishnan et al., 2009). Coomassie stained gels were destained with multiple changes of ultrapure water to remove background and scanned using an Epson Perfection V700 scanner controlled through Adobe Photoshop. Images were analyzed for proteome differences using Delta2D image analysis software. Delta2D parameters were set to maximize spot detection using global image warping and exact spot matching. Background subtraction parameters were identical for all gels and eliminated a large percentage of spots that were less than 0.05% of the total normalized spot volumes. A total of 732 remained on the fusion image of all gels included in the analysis, after background subtraction, and were used for the total volume ratio normalization. A total of 34 spots were chosen as a subset of this total, for major seed storage protein quantification, comprising nearly 50% of the total volume ratio normalized spot volume. This subset of 2-D resolved proteins were previously identified (Krishnan et al., 2009; Krishnan and Nelson, 2011) using peptide mass fingerprinting (MALDI-TOF MS) and then categorized as shown in **Figure 5**. The exact same spot regions were chosen from software generated fused image of all gels used in the analysis. Spot % volume quantities were calculated within each comparison individually; Maverick (*n* = 3) and SAM (*n* = 3).

### Sulfur treatment and plant growth conditions

Soybeans were grown in an environmentally controlled growth chamber at 26 ± 2°C and 50% humidity. The plants received 14 h of daylight for the duration of the experiment. Each individual plant was grown in its own modified hydroponics container, consisting of a pot containing a 1:1 mixture of perlite:vermiculite, with a lower 2 L reservoir for the nutrient solution. Cotton wicks were placed in the lower portion of each pot to deliver nutrient solution to the plant prior to the root growth eventually reaching the lower reservoir. The nutrient solution composition was: 1.25 mM Ca(NO_3_)_2_ 4H_2_O, 1.25 mM KNO_3_, 0.25 mM KH_2_PO_4_, 0.5 mM Fe-EDTA, 0.5 mM NH_4_NO_3_, and a 1/1000th addition of a micronutrients mixture (H_3_BO_3_, 2.86 g L^−1^; MnCl_2_ 4H_2_O, 1.81 g L^−1^; ZnCl_2_, 0.095 g L^−1^; Cu(NO_3_)_2_ xH_2_O, 0.047 g L^−1^; H_2_MoO 4H_2_O, 0.09 g L^−1^). The sulfate control solution contained 0.5 mM MgSO_4_ 7H_2_O, and the sulfur-rich solution contained 2.0 mM MgSO_4_ 7H_2_O. The nutrient solution was replaced every 3 days to maintain a consistently fresh nutrient supply. Seeds were harvested approximately 90 days after treatments began.

### Amino acid analysis

Amino acid analysis was performed at the Donald Danforth Plant Science Center Proteomics and Mass Spectrometry Facility. Free amino acid content was determined following the protocol of Hacham et al. (2002). For the determination of the total amino acid content the seed powder was first subjected to hydrolysis with 6N HCl. For the quantification of methionine and cysteine, duplicate samples were first subjected to an initial oxidation step using performic acid prior to acid hydrolysis. Amino acids were quantified using the manufacturer's instructions of the Waters AccQ-Tag Ultra Kit on an Acquity UPLC system. Samples were run in quadruplicate and subjected to appropriate statistical analysis.

### Immunostaining of paraffin sections

Greenhouse-grown soybean seeds at R6 stage (Fehr et al., 1971) were cut into several pieces and fixed in FAA (10% formaldehyde, 50% ethyl alcohol, and 5% glacial acetic acid) for 8 h at room temperature. The tissue was dehydrated in a graded ethanol/xylene series and infiltrated with paraffin. Sections were cut with a microtome and processed for immunostaining following the protocol described earlier (Bilyeu et al., 2008). Sections were separately incubated with 1:1000 diluted antibodies raised against the β-subunit of β-conglycinin or Kunitz trypsin inhibitor. Following this step, the sections were treated sequentially with biotinylated linker, streptavidin conjugated to horseradish peroxidase, and substrate-chromogen solution (DAKO). The sections were examined under bright field optics.

### Electron microscopy

Mature dry seeds were surface sterilized by first sequentially incubating with 95% ethanol for 5 min and with 50% commercial bleach for 5 min. Following extensive washes in distilled water the seeds were transferred to 1% water agar plates and germinated in a 30°C incubator for 12 h. Seeds were sliced into several 2–4 mm cubes and fixed for 4 h in 2.5% glutaraldehyde buffered at pH 7.2 with 50 mM sodium phosphate. After several rinses in sodium phosphate buffer the seeds were post-fixed for 1 h with 1% aqueous osmium tetroxide. The seed tissue was dehydrated in a graded acetone series and infiltrated with Spurr's resin. Thin sections of the seed tissue were cut with a diamond knife and collected on 200 mesh copper grids. The grids were stained with 0.5% uranyl acetate and 0.4% lead citrate and examined at 80 kV under JEOL 1200 EX (Tokyo, Japan) transmission electron microscope.

## Results

### Creation of B-conglycinin knockdown soybean seeds

**Figure 2:**
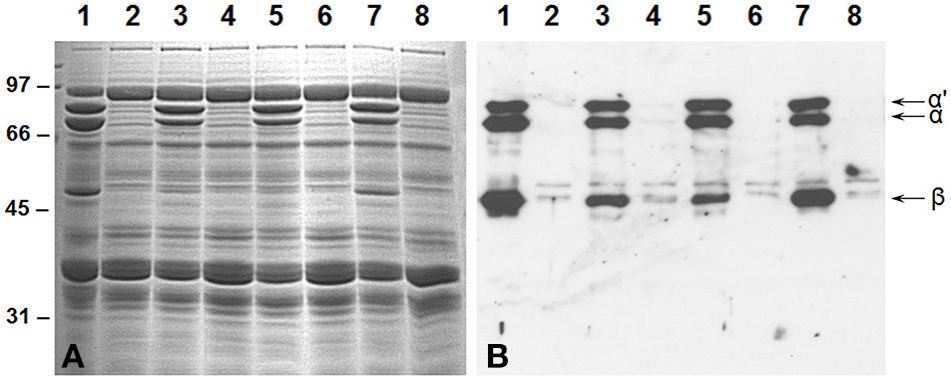
**Suppression of β-conglycinin accumulation in transgenic soybean lines**. Total seed proteins from wild-type (lane 1) and from seven independent RNAi transgenic lines (lanes 2–8) were fractionated in duplicate gels by 10% SDS-PAGE and stained with Coomassie Blue **(A)** or transferred to nitrocellulose membranes. The membrane was probed with the β-conglycinin antibodies **(B)**. Immunoreactive proteins were identified using anti rabbit IgG-horseradish peroxidase conjugate followed by chemiluminescent detection. Note the absence of α′, α, and β-subunits of β-conglycinin in some of the RNAi lines.

The suppression of the 7S globulins accumulation in soybean seeds was accomplished by RNAi utilizing *Agrobacterium*-mediated cotyledonary node transformation protocol (Hinchee et al., 1988). Seeds from several independent transgenic lines were screened for their protein composition by SDS-PAGE and immunoblot analysis using antibodies raised against the β-subunit of β-conglycinin (Figure 2). An examination of the Coomassie stained gel revealed that in 50% of the transgenic plants no significant reduction in the accumulation of β-conglycinin had occurred when compared to the wild-type plant (Figure 2A). However, four transgenic RNAi lines showed a complete absence of the β-conglycinin accumulation in their seeds (Figure 2A). The lack of β-conglycinin in these RNAi lines was confirmed by immunoblot analysis using β-conglycinin antibodies (Figure 2B). These four lines were grown for five generations. Twenty individual T5 seeds from each transgenic line were subjected to Western blot analysis. Transgenic plants where all tested seeds failed to accumulate the β-conglycinin were considered to be homozygous plants. These homozygous transgenic RNAi plants are referred as SAM from this point onwards.

**Figure 3:**
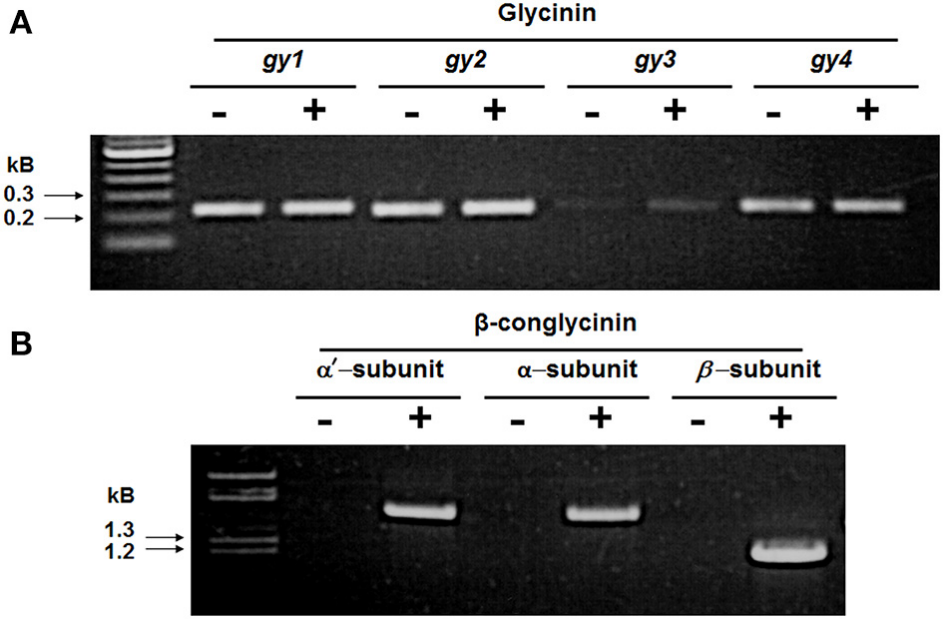
**RT-PCR analysis of 7S and 11S globulin gene expression in developing soybean seeds**. Total RNA isolated from wild-type control (+) and β-conglycinin knockdown transgenic soybean line SAM (−) was used in RT-PCR analysis. The upper panel **(A)** shows the RT-PCR products for the *gy1*, *gy2*, *gy3*, and *gy4* genes (11S globulin) and the lower panel **(B)** shows the RT-PCR products for the α′, α, and β-subunits of β-conglycinin (7S globulin).

We investigated the mRNA levels of the major seed storage proteins between the RNAi line SAM and the wild-type control by RT-PCR. When primers specific for *gy*1, *gy*2, *gy*3, and *gy*4 were used in RT-PCR reactions, gene-specific products were amplified in both plants (Figure 3A). However, when primers specific for each of the three subunits of β-conglycinin were employed, RT-PCR products were seen only in the wild-type control and not in SAM (Figure 3B). The absence of the β-conglycinin accumulation in SAM seeds was further examined by immunocytochemical localization. Sections of soybean seed were incubated with either β-conglycinin or Kunitz trypsin inhibitor specific antibodies followed by incubation with streptavidin conjugated to horseradish peroxidase. Antibodies raised against the β-conglycinin did not detect their accumulation in the paraffin-embedded sections as evidenced by the absence of brown localization signal (Supplemental Figure 1A) while it was readily detected in the wild-type control seeds (Supplemental Figure 1B). In contrast, when the paraffin sections were incubated with Kunitz trypsin inhibitor specific antibodies positive reactions were seen in both SAM and the wild-type control seeds (Supplemental Figures 1C–D).

### Protein, oil and amino acid composition of B-conglycinin knockdown soybean seeds

To investigate if knockdown of β-conglycinin resulted in any alterations of the seed components we first measured the total protein and oil content of these seeds. Both the wild-type control and SAM contain very similar concentrations of total protein and oil (Table 1). Total amino acid content was significantly higher in β-conglycinin knockdown line (Table 1). Analysis of the five major fatty acids in these seeds by gas chromatograph also showed significant differences. The concentration of palmitic, linoleic and linolenic acids was higher in the wild-type while oleic acid content was higher in SAM (Table 1).

**Table 1:** **Protein, amino acid and oil content in wild-type and β-conglycinin knockdown line (SAM)**.

**Seed Component**	**Wild-type**	**SAM**	**Significance level**	***p*-value**
Protein^a^	35.9 ± 0.4	36.0 ± 0.4	NS	0.84
Oil^a^	19.4 ± 0.1	19.7 ± 0.3	NS	0.05
Total Amino Acid (Free + Protein bound)^b^	2410.5 ± 113.4	2794.5 ± 130.1	^**^	4.53E-03
Fatty acids^a^				
Palmitic Acid (16:0)	11.6 ± 0.2	10.6 ± 0.1	^***^	3.76E-04
Stearic Acid (18:0)	4.2 ± 0.1	4.5 ± 0.3	NS	0.08
Oleic Acid (18:1)	20.5 ± 0.9	23.2 ± 0.7	^**^	3.38E-03
Linoleic Acid (18:2)	55.9 ± 0.6	55.0 ± 0.4	^*^	0.02
Linolenic Acid (18:3)	7.9 ± 0.2	6.7 ± 0.1	^***^	6.25E-04

Significantly different means are indicated by ^*^, ^**^, or ^***^ (p ≤ 0.05, p ≤ 0.01, p ≤ 0.001, respectively) and insignificant differences are indicated by “NS.”

a expressed as percentage.

b nanomoles/mg seed.

**Figure 4:**
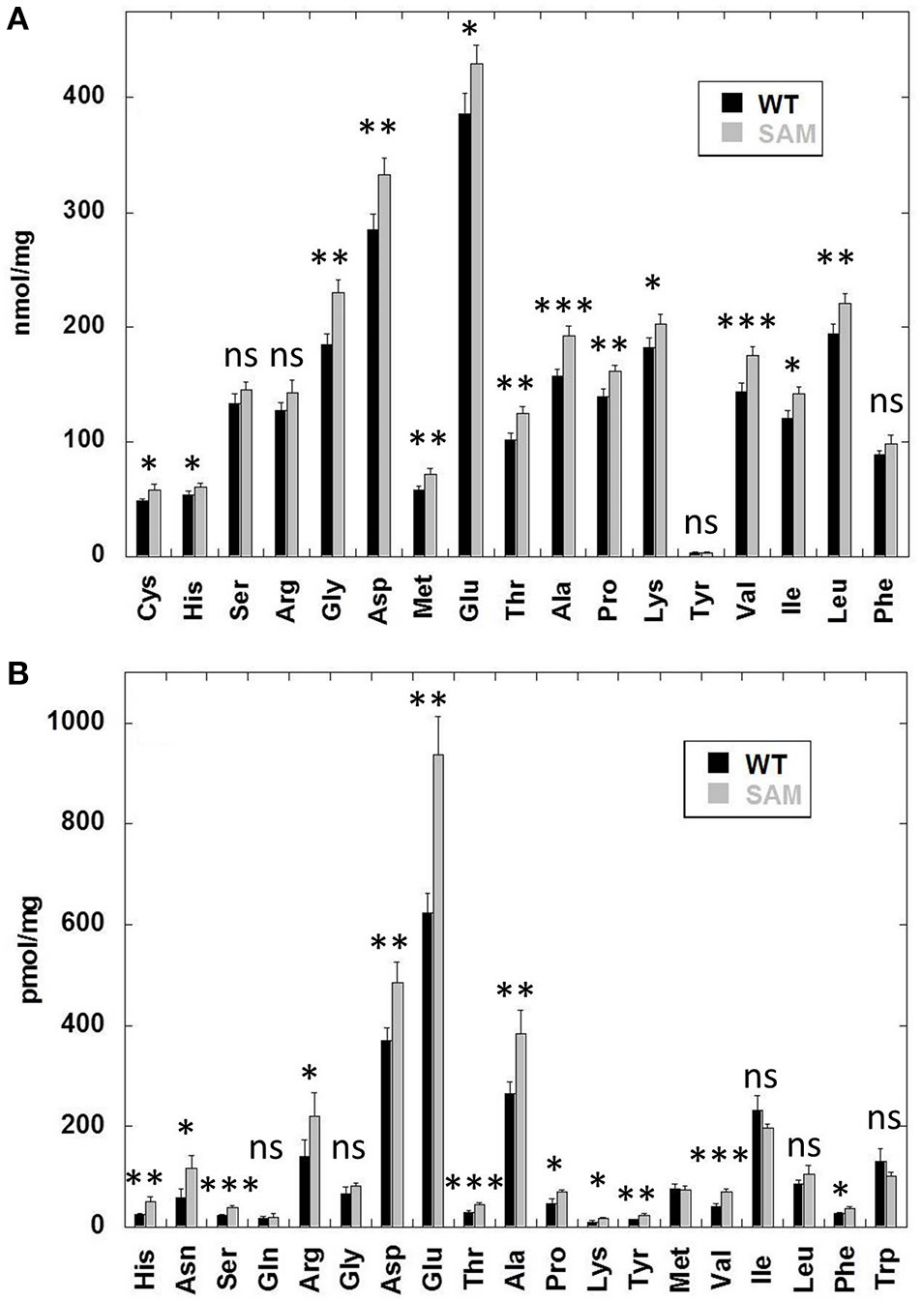
**Free and total amino acid composition of soybean seeds**. Dry seed powder from quadruplicate samples was analyzed by HPLC to measure the total **(A)** and free amino acid **(B)** from wild-type (WT) and β-conglycinin knockdown soybean line (SAM).

Previous studies have shown that soybean mutants lacking the seed storage proteins accumulate high levels of free amino acids (Takahashi et al., 2003; Schmidt et al., 2011). To examine if similar situation also occurred in the β-conglycinin knockdown soybean line we determined the total and free amino acid content (Figure 4). An examination of the total amino acid composition revealed a slight increase in several of the amino acids in SAM when compared to that of wild-type control (Figure 4A). In contrast, a comparison of the free amino acid composition revealed significant increases in the concentration of several amino acids in SAM seeds especially glutamic acid, aspartic acid, asparagine, arginine, and alanine (Figure 4B). In previous studies arginine represented the main amino acid that contributed to the accumulation of high levels of free amino acids (Takahashi et al., 2003; Schmidt et al., 2011). However, in our studies we observed that arginine was not the most abundant amino acid in the overall free amino acid pool in SAM. Few other amino acids (glutamic acid, aspartic acid, asparagine, and alanine) also contributed significantly to the elevated levels of free amino acid content in SAM seeds (Figure 4B).

### 2D-gel analysis of soybean seed protein composition

**Figure 5:**
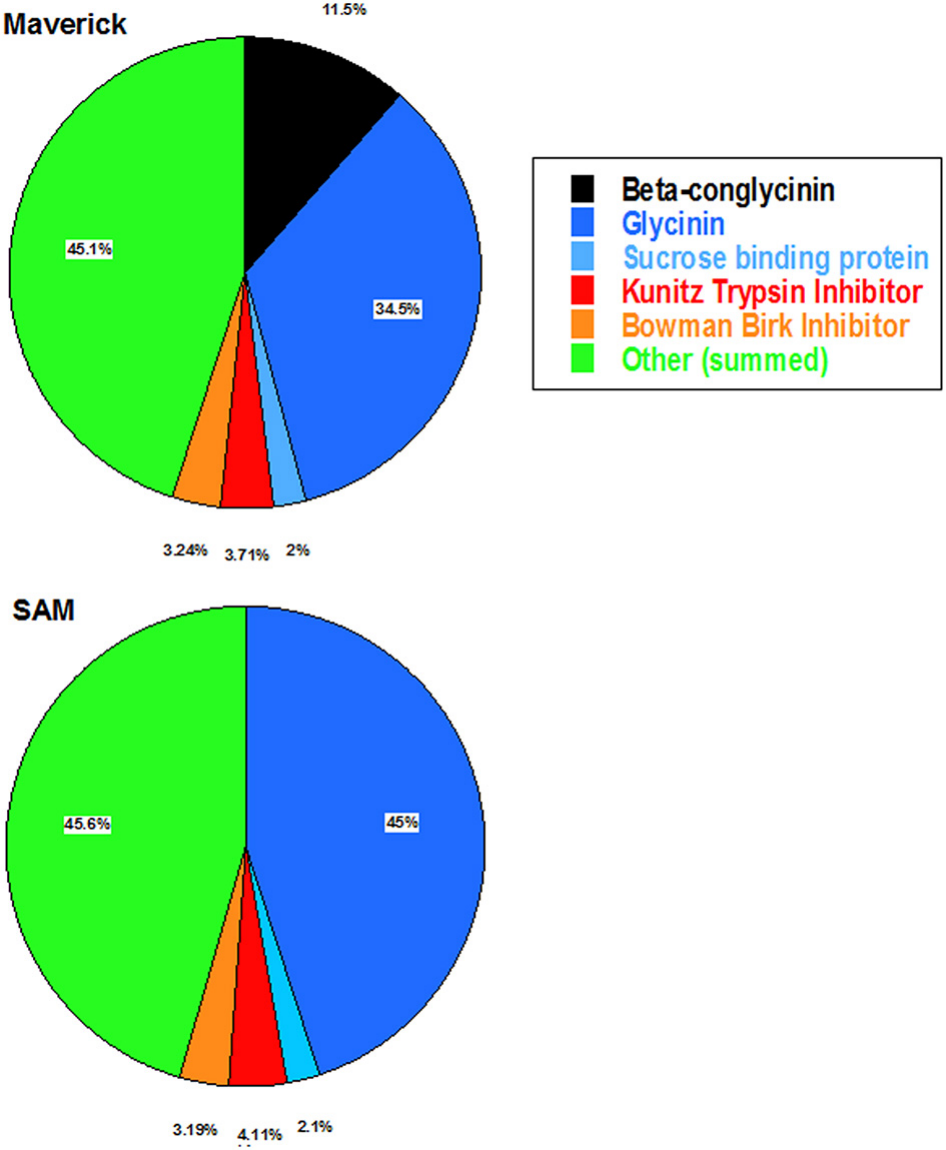
**Pie chart representation of the abundance of proteins in the proteome of Maverick (wild-type) and β-conglycinin knockdown soybean line (SAM)**. Equal amounts of proteins from soybean seeds were analyzed by high-resolution 2D gel electrophoresis. Spot volume measurements of the seed proteins averaged from triplicate 2-D gels were determined by using Delta2D image analysis software. The pie chart demonstrates that the suppression of the β-conglycinin in SAM results in increased accumulation of the glycinin.

Two dimensional-gel analysis was performed to evaluate the changes in the protein composition of RNAi soybean line with that of the wild-type control (Figure 5). This analysis clearly demonstrated that β-conglycinin knockdown RNAi line lacked all the three subunits (α′, α, and β-subunits) of β-conglycinin. Previous studies have shown that suppression of the seed 7S and 11S seed storage proteins resulted an increase in the accumulation of lipoxygenase, sucrose binding protein, basic 7S globulin, Kunitz trypsin inhibitor, soybean lectin, Gly m Bd 30k, Glc-binding protein and seed maturation-associated protein in these seeds (Takahashi et al., 2003; Schmidt et al., 2011). To see if similar changes are also occurred in SAM 2D fractionated soybean seed proteins from SAM and wild-type control (run in triplicate) were analyzed with high-resolution image analysis software (Delta 2D). This analysis indicated that absence of the three subunits of β-conglycinin was associated with an increase in the glycinin content (Figure 5). A comparison of seed protein profiles between SAM and wild-type control did not reveal any substantial differences between other seed proteins (Figure 5).

### Production of transgenic soybean lines expressing the 11 kDa δ-zein and it's introgression into SAM

**Figure 6:**
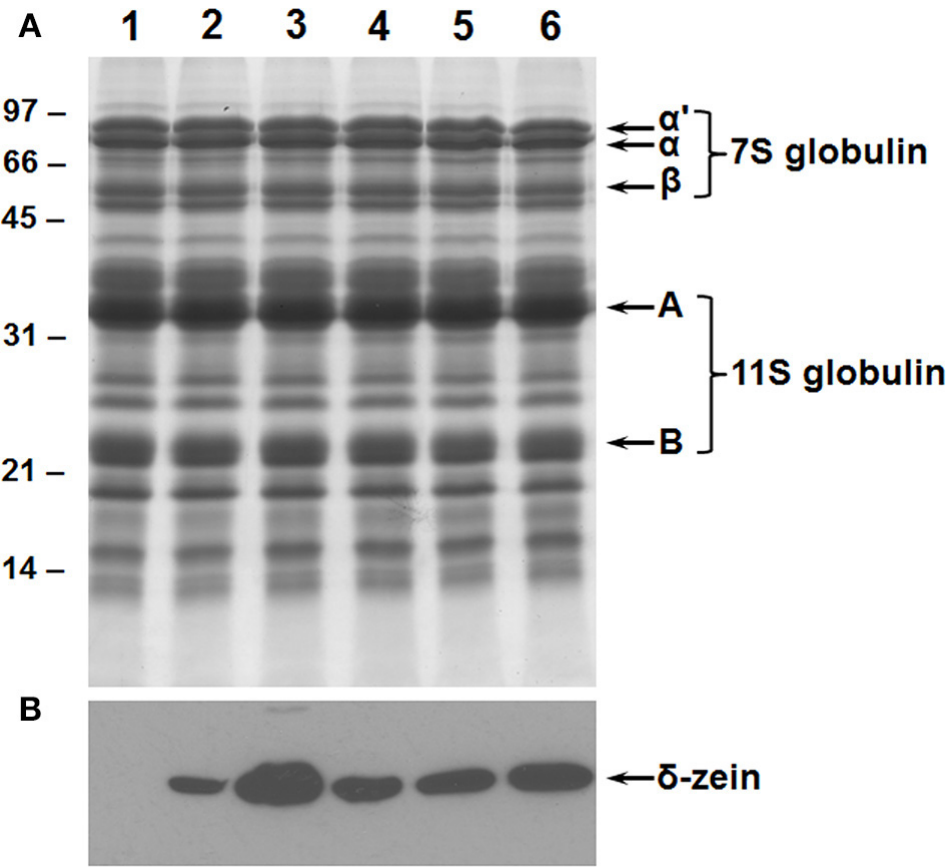
**Accumulation of 11 kDa delta zein in transgenic soybean seeds**. Total seed proteins from dry soybean seeds from wild type (lane 1) and five independent transgenic lines (lanes 2–6) expressing the 11 kDa δ-zein under the control of glycinin promoter were resolved on a 15% SDS-PAGE. The gels were either stained with Coomassie Brilliant Blue **(A)** or subjected to western blot analysis using maize 11 kDa delta zein antibodies **(B)**. Immunoreactive proteins were detected by chemiluminescent method. Sizes of protein standards are shown in kilodaltons.

We had earlier generated transgenic soybean plants expressing 11 kDa δ-zein under the control of the β-conglycinin promoter (Kim and Krishnan, 2004). Since the β-conglycinin promoter was also used for suppressing the expression of β-conglycinin we wanted to use another seed-specific promoter to express the 11 kDa δ-zein. For this purpose a plasmid consisting of soybean glycinin promoter, the coding region of the 11 kDa δ-zein, the 3′ region of the potato proteinase inhibitor gene, together with the cassette containing *bar* herbicide resistance gene was constructed (Figure 1) and introduced into soybean cv. Williams 82 as described earlier (Kim and Krishnan, 2004). The accumulation of the 11 kDa δ zein in five independent transgenic events was verified by western blot analysis using antibodies raised against the purified 11 kDa δ-zein (Figure 6). The 11 kDa δ-zein expressing soybean lines were grown in the greenhouse for another three generations to produce T4 plants.

Earlier studies have shown proteome rebalancing in seeds can be exploited for elevated expression of foreign proteins (Goossens et al., 1999; Tada et al., 2003; Schmidt and Herman, 2008). We wanted to test if the δ-zein introgression into the β-conglycinin knockdown RNAi line would enhance the accumulation of the δ-zein. For this purpose, crosses were made between homozygous 11 kDa δ-zein expressing soybean plants and β-conglycinin knockdown RNAi soybean plants. Seeds from successful crosses were germinated and a small segment of the seed was assayed by immunoblot analysis for the absence of β-conglycinin and the accumulation of 11 kDa δ-zein. Seeds with the desired combination were grown in the green house and subjected to recurrent selection until homozygous plants with the desired traits were obtained.

The accumulation of the 11 kDa δ-zein in the β-conglycinin knockdown RNAi soybean plant was examined by immunoblot analysis. The 11 kDa δ-zein was readily detected in both original transgenic soybean plants and in β-conglycinin knockdown RNAi background. However, the 11 kDa δ-zein did not accumulate at higher amounts in soybean seeds lacking β-conglycinin, indicating that proteome rebalancing did not enhance the accumulation of the 11 kDa δ-zein.

**Figure 7:**
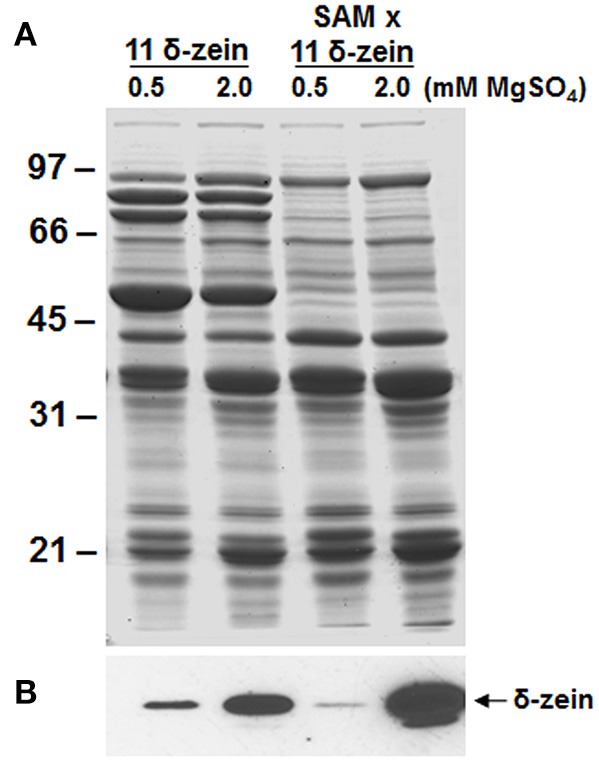
**Effect of sulfur nutrition on the accumulation of the 11 kDa δ-zein in transgenic soybean seeds**. Total seed proteins from 11 kDa δ-zein expressing line and the 11 kDa δ-zein introgressed into the β-conglycinin knockdown soybean line (SAM) that were grown hydroponically in presence of 0.5 or 2 mM magnesium sulfate were fractionated in duplicate gels by 15% SDS-PAGE and stained with Coomassie Blue **(A)** or transferred to nitrocellulose membranes. The membrane was probed with the 11 kDa δ-zein specific antibodies **(B)**. Immunoreactive proteins were identified using anti rabbit IgG-horseradish peroxidase conjugate followed by chemiluminescent detection.

It has been shown that availability of methionine and cysteine in legumes is the major limiting factor in enhancing the sulfur content of legumes (Tabe et al., 2002). We therefore, examined if the accumulation of the 11 kDa δ-zein can be promoted by providing the developing plants with sulfate-rich solution, which can be assimilated for sulfur metabolism. For this purpose, we grew the soybean plants in hydroponics under two levels of sulfate. In seeds from plants grown in presence 0.5 mM magnesium sulfate, the accumulation of the 11 kDa δ-zein was higher in wild-type background than in β-conglycinin knockdown background (Figures 7A–B). Interestingly, in seeds from soybean plants grown in presence of 2 mM magnesium sulfate there was a drastic increase in the accumulation of the 11 kDa protein. This difference in the accumulation of the δ-zein was much more striking in β-conglycinin knockdown background (Figure 7B). Densitometer scans of the immunoblots revealed that the seeds from plants grown in presence of 2 mM magnesium sulfate had about 3- to 16-fold increases in the accumulation of the 11 kDa δ-zein in the original and introgressed lines, respectively (Figure 7B). In contrast, the overall protein content of soybean seeds was not affected by sulfate treatment. Soybean plants grown in presence of 2 mM magnesium sulfate had 34.9 ± 0.6% while those grown in sulfate-rich solution contained 35.0 ± 0.4% protein. Our preliminary analysis also revealed that the transgenic soybean plants grown in sulfate-rich solution exhibited 1.2 fold-increase in the total sulfur amino acid content when compared with plants grown in presence of 0.5 mM magnesium sulfate.

### Sulfur supplementation promotes the formation of protein bodies

**Figure 8:**
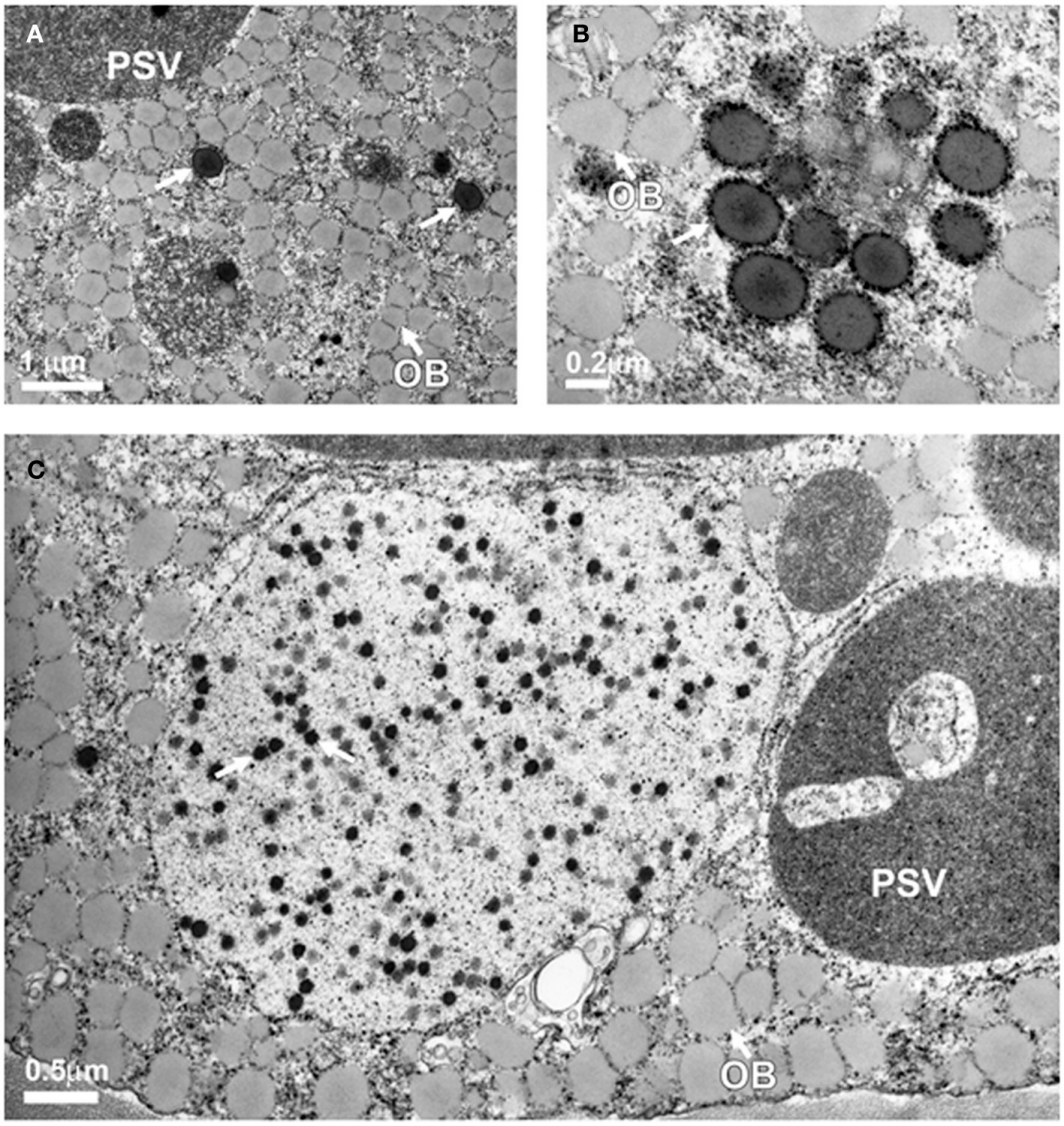
**Transmission electron microscopy observation of protein bodies in transgenic soybean seeds**. Seeds grown in presence of 0.5 mM magnesium sulfate contain a few endoplasmic reticulum-derived spherical protein bodies (**A**, arrows) while seeds grown in presence of 2 mM magnesium sulfate reveal numerous dark staining spherical protein bodies **(B,C)**. PSV, protein storage vacuole; OB, oil bodies; PB, protein body.

Previously we showed that expression of 11 kDa δ-zein results in the formation of endoplasmic reticulum derived protein bodies (Kim and Krishnan, 2004). Immunocytochemical localization studies also confirmed that δ-zein is localized within these protein bodies (Kim and Krishnan, 2004). Since sulfur supplementation resulted in a drastic increase in the accumulation of the 11 kDa δ-zein in transgenic soybean plants, we examined if this change was accompanied by an increase in the number of protein bodies. Thin-sections of soybean seeds expressing the 11 kDa δ-zein in β-conglycinin knockdown background grown in 0.5 and 2 mM of magnesium sulfate were examined by transmission electron microscopy (Figure 8). Electron microscopy observation of thin sections of soybean seeds grown in presence of 0.5 mM magnesium sulfate revealed prominent oil bodies and large protein storage vacuoles, the storage compartment for the native glycinin and β-conglycinin (Figure 8A). In addition few spherical electron-dense spherical protein bodies were also observed in the cotyledonary cells (Figure 8A). An examination of seeds grown in 2 mM of magnesium sulfate revealed the presence of numerous protein bodies (Figure 8B). Interestingly soybean seeds from plants grown in presence of 2 mM magnesium sulfate contained numerous small electron-dense protein bodies within vacuoles (Figure 8C).

## Discussion

Soybeans provide several advantages for the production of recombinant proteins for diagnostics, therapeutics, and industrial applications (Bost and Piller, 2011). Chief among them is the low cost of recombinant protein production and the ability to produce and store large amounts of transgenic proteins in seeds (Bost and Piller, 2011). Several laboratories have generated transgenic soybean plants that express 1 to 4% of the total soluble protein as recombinant protein (Piller et al., 2005; Ding et al., 2006; Garg et al., 2007; Moravec et al., 2007; Rao and Hildebrand, 2009; Cunha et al., 2010). Other groups have identified seed fill sensor proteins, such as cruiferin, that may be usable to alter seed composition (Lin et al., 2013). In spite of these achievements, problems remain in obtaining and maintaining high-level heterologous expression in seeds. In cases where high-level expression of transgene is achieved, invariably it is accomplished by down regulation of endogenous reserve proteins (Tabe and Droux, 2001; Hagan et al., 2003; Scossa et al., 2008).

Several naturally occurring, as well as induced, mutations that affect the accumulation of soybean seed storage proteins have been previously described (Kitamura and Kaizuma, 1981; Odanaka and Kaizuma, 1989; Takahashi et al., 1994; Yagasaki et al., 1996; Hayashi et al., 1998). By integrating these mutations by crossbreeding, a soybean line that lacks all the glycinin and β-conglycinin subunits has been developed (Takahashi et al., 2003). This soybean line, in spite of lacking all the major seed storage proteins, was able to grow and reproduce normally. Interestingly, the nitrogen content of the seeds was found to be similar to that of the wild-type cultivars (Takahashi et al., 2003). Additionally, the absence of the abundant seed proteins resulted in preferential increase in the accumulation of lipoxygenase, sucrose binding protein, agglutinin and the basic 7S globulin. Similarly, a soybean line lacking both glycinin and beta-conglycinin (SP-) was developed by RNA interference (Schmidt et al., 2011). The absence of glycinin and β-conglycinin in the SP- line was accompanied by selective increase in the accumulation of a few proteins similar to the situation encountered through integration of mutations (Takahashi et al., 2003). In both cases, the absence of major seed storage proteins was compensated by the accumulation of free amino acids with arginine accounting for more than 50% of the free amino acid content (Takahashi et al., 2003; Schmidt et al., 2011).

Here we used RNAi to suppress expression of the α, α′, and β-subunits of β-conglycinin in soybean seed. The resulting SAM line showed clear knockdown of these subunits, as confirmed by RT-PCR, immunoblot, and 2D-gel analyses (Figures 2, 3, 5). Although the SAM line retained normal overall protein and oil seed content compared to wild-type seed (Table 1), the distribution of amino acids in SAM showed increases in glutamic acid, aspartic acid, asparagine, and alanine compared to wild-type (Figure 4). In contrast to Takahashi et al. (2003), only a modest increase in arginine content in the β-conglycinin knockdown soybean line was observed (Figure 4). Moreover, this comparison revealed slight increase in the methionine and cysteine content in the β-conglycinin knockdown soybean line compared to wild-type. Glycinin is relatively rich in sulfur, while the β-conglycinin is poor in sulfur containing amino acids (Krishnan, 2005). Some earlier reports suggest that elimination of β-conglycinin could led to increased levels of sulfur-containing amino acids in soybean seeds (Ogawa et al., 1989; Panthee et al., 2004). Our analysis of the SAM seeds indicates that elimination of β-conglycinin only marginally increases the sulfur amino acid content of soybean seeds. This observation is consistent with other studies indicating that a lack of either glycinin or β-conglycinin has little effect on total amino acid composition of soybean seeds (Takahashi et al., 2003; Schmidt et al., 2011).

RNA interference has been successfully employed to alter the nutritional quality of the seed (Segal et al., 2003; Frizzi et al., 2010). For example, the suppression of the barley C-hordeins resulted in elevated levels of several essential amino acids in the transgenic seeds (Lange et al., 2007). Similarly, high-level expression of human growth hormone polypeptide was achieved in a glutelin and prolamin knockdown rice line (Shigemitsu et al., 2012). In contrast, our approach to increase the accumulation of the methionine-rich maize δ-zein in the β-conglycinin knockdown soybean line showed no appreciable changes in expression levels of the maize protein.

Previous attempts to overexpress foreign proteins in seed protein knockdown soybean lines have also met with mixed results. The introgression of green fluorescent protein (GFP-kdel) in a β-conglycinin suppressed soybean line resulted in a four-fold increase in the accumulation of the GFP-kdel (Schmidt and Herman, 2008). Subsequently, researchers from the same group reported that introgression of GFP in a glycinin and β-conglycinin knockdown (SP-) line did not lead to accumulation of GFP (Schmidt et al., 2011). The results from these studies clearly indicates that the capacity to overexpress foreign proteins in soybeans will be influenced by several factors such as the nature of the endogenous seed protein targeted for suppression by RNAi, the amino acid composition, and the localization of the heterologous protein.

An obstacle in achieving high-level expression of methionine-rich δ-zein may be the paucity of sulfur-containing amino acids in developing soybean seeds to support its production. Legumes in general are poor in sulfur-containing amino acids (Shewry, 2000). Often the expression of foreign proteins rich in sulfur amino acids is accompanied by reduction in the endogenous sulfur-rich proteins, indicating that sulfur availability in seeds is the limiting factor. Sulfur nutrition or methionine supplementation has been shown to influence the accumulation of methionine-rich storage proteins in legumes (Chiaiese et al., 2004; Amira et al., 2005). Common bean (*Phaseolus vulgaris* L.) accumulates large amounts of a γ-glutamyl dipeptide of S-methyl-cysteine, a non-protein amino acid (Taylor et al., 2008). Interestingly, the removal of 7S globulin and phytohemagglutinin in *Phaseolus vulgaris* enhanced the accumulation of sulfur-rich proteins (Marsolais et al., 2010). Additionally, an increase in cysteine and methionine was reported which occurred at the expense of S-methylcysteine. This indicates a redirection of sulfur from γ-glutamyl-S-methyl-cysteine to the protein cysteine pool.

When the transgenic soybean seeds were grown in presence of excess sulfur there was a significant increase in the accumulation of the methionine-rich δ-zein (Figure 7). Transmission electron microscopy observation (Figure 8) also confirmed that sulfur supplementation to soybean plants drastically increased the number of endoplasmic reticulum derived protein bodies, the site of δ-zein accumulation. These observations indicate that the intrinsic capacity to synthesize sulfur containing amino acids is very high but limited by the level of sulfate in the nutrient solution. Previous studies indicate that metabolic engineering of the sulfur assimilatory pathway can be used as a tool to increase the sulfur amino acid content of seeds (Avraham et al., 2005; Tabe et al., 2010; Song et al., 2013). Recently, it was reported that soybean seeds expressing feed-back-insensitive cystathionine-γ-synthase exhibited 1.8 to 2.3-fold increases in the total methionine content of their seeds (Song et al., 2013).

In this study we have shown the importance of sulfur nutrition on the accumulation of heterologous methionine-rich protein in soybean. Our results suggest that sink strength is not limiting for accumulation of methionine-rich δ-zein. Instead the limiting factor appears to be sulfate availability. Sulfur assimilation is inter-connected with nitrogen (N) and carbon (C) metabolism (Kopriva et al., 2002; Hawkesford and De Kok, 2006). Because of this inter-connection sulfur deficiency manifests in the form of poor plant growth and lower yields (Zhao et al., 1999a,b; Hawkesford, 2000). Insufficient sulfur supply causes changes in amino acid pools and alters the seed protein composition (Gayler and Sykes, 1985; Spencer et al., 1990). Additionally, sulfur nutrition has a pronounced effect on legume-rhizobium symbiosis. Legumes grown in sulfur-rich media exhibit elevated nitrogen fixation due to an increase in nodule development and function (Scherer et al., 2008; Varin et al., 2010). Compared to other organs nodules have much greater thiol concentrations due to active thiol synthesis in nodule tissue (Matamoros et al., 1999). Higher yields observed in legumes grown in sulfur-rich environment may be attributed to remobilization of nutrients from the nodules and other source organs to seeds. In a recent study, it was demonstrated that the response of plants to sulfur deficiency is dependent on the developmental stage of the plant (Zuber et al., 2013). Sulfur deficiency imposed at the mid-developmental stage of a model legume, *Medicago truncatula*, decreased yield and altered the allocation of nitrogen and carbon to seeds (Zuber et al., 2013). Interestingly, sulfur deficiency imposed during the reproductive period had little influence on the yield and nutrient allocation (Zuber et al., 2013).

Elevating the accumulation of sulfur-rich proteins in legumes possesses unique challenges. Recently, we reported a successful strategy to increase the sulfur amino acid content of soybean seed proteins by overexpressing a cytosolic isoform of *O*-acetylserine sulfhydrylase (Kim et al., 2012). These transgenic soybean plants contained elevated levels of sulfur-containing amino acids that promoted the accumulation of Bowman-Birk protease inhibitor, a cysteine-rich protein (Kim et al., 2012). Thus, introgression of foreign proteins rich in sulfur-containing amino acids into the *O*-acetylserine sulfhydrylase overexpressing soybean line may offer a viable strategy to increase the sulfur amino acid content of soybean seeds. It is worthwhile to note that co-transformation of potato plants with methionine insensitive cystathionine γ-synthase (CgS_Δ90_) and 15 kD β-zein resulted in elevation of protein-bound methionine content (Dancs et al., 2008).

## Author contributions

Won-Seok kim, Joseph M. Jez and Hari B. Krishnan designed research; Won-Seok kim and Hari B. Krishnan performed research; Hari B. Krishnan and Joseph M. Jez analyzed the data and wrote the paper.

### Conflict of interest statement

The authors declare that the research was conducted in the absence of any commercial or financial relationships that could be construed as a potential conflict of interest.
