# Supplementary figures and images for: Effects of proteome rebalancing and sulfur nutrition on the accumulation of methionine rich δ-zein in transgenic soybeans
Source: Front Plant Sci. 2014 Nov 11;5:633. doi: 10.3389/fpls.2014.00633 (PMC4227475; doi:10.3389/fpls.2014.00633)

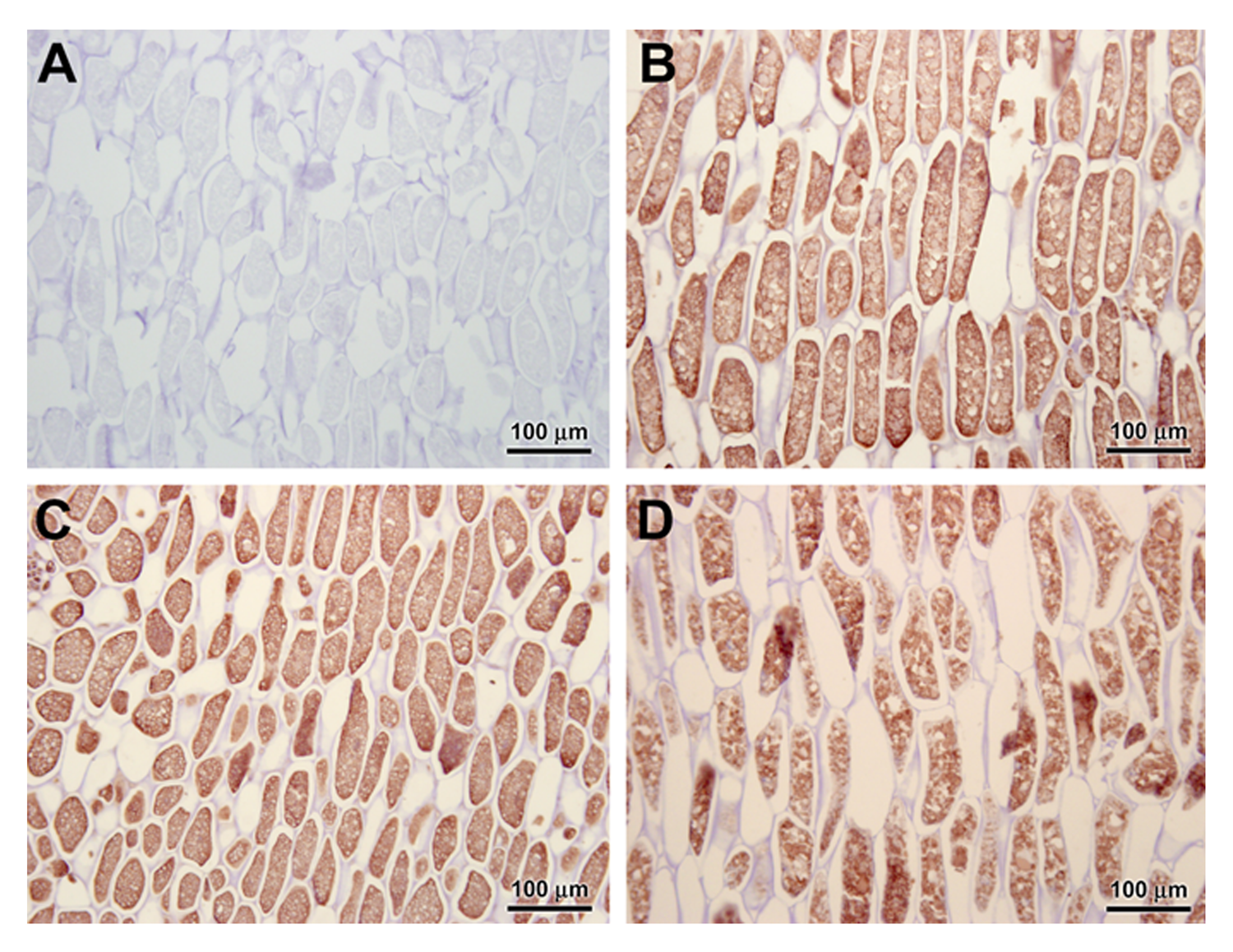

Supplement: Supplementary file 1 [file Image1.TIF]
